# Synergy of Nanotopography and Electrical Conductivity of PEDOT/PSS for Enhanced Neuronal Development

**DOI:** 10.1021/acsami.3c15278

**Published:** 2023-12-13

**Authors:** Michele Bianchi, Sonia Guzzo, Alice Lunghi, Pierpaolo Greco, Alessandra Pisciotta, Mauro Murgia, Gianluca Carnevale, Luciano Fadiga, Fabio Biscarini

**Affiliations:** † Department of Life Sciences, Università degli Studi di Modena e Reggio Emilia, 44125 Modena, Italy; ‡ Center for Translational Neurophysiology of Speech and Communication, Istituto Italiano di Tecnologia, 44121 Ferrara, Italy; § Section of Physiology, Università di Ferrara, 44121 Ferrara, Italy; ∥ Department of Surgery, Medicine, Dentistry and Morphological Sciences with Interest in Transplant, Oncology and Regenerative Medicine, Università di Modena e Reggio Emilia, 44125 Modena, Italy; ⊥ Istituto per lo Studio dei Materiali Nanostrutturati (ISMN-CNR), 40129 Bologna, Italy

**Keywords:** surface nanomodulation, electrical stimulation, atomic force microscopy, neural cells, neurite outgrowth

## Abstract

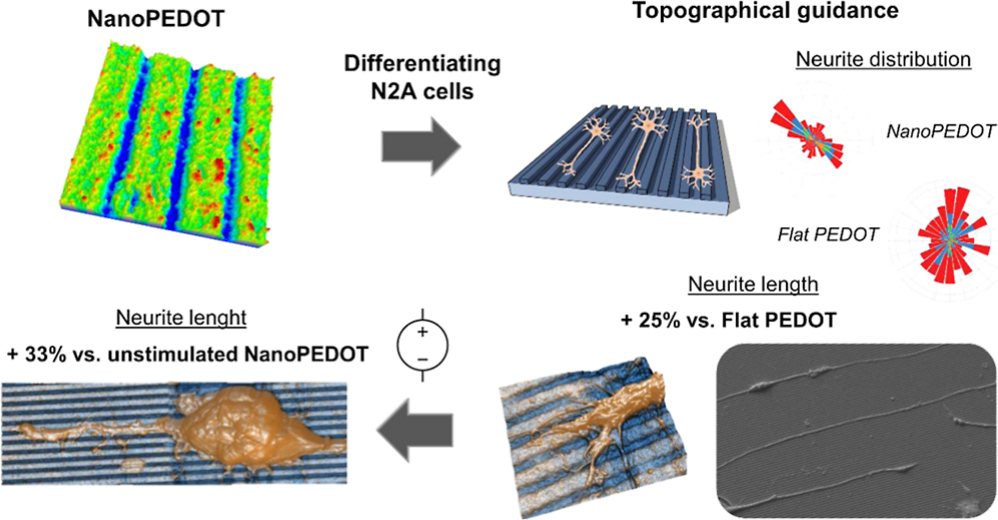

Biomaterials able to promote neuronal development and neurite outgrowth are highly desired in neural tissue engineering for the repair of damaged or disrupted neural tissue and restoring the axonal connection. For this purpose, the use of either electroactive or micro- and nanostructured materials has been separately investigated. Here, the use of a nanomodulated conductive poly(3,4-ethylendioxithiophene) poly(styrenesulfonate) (PEDOT/PSS) substrate that exhibits instructive topographical and electrical cues at the same time was investigated for the first time. In particular, thin films featuring grooves with sizes comparable with those of neuronal neurites (NanoPEDOT) were fabricated by electrochemical polymerization of PEDOT/PSS on a nanomodulated polycarbonate template. The ability of NanoPEDOT to support neuronal development and direct neurite outgrowth was demonstrated by assessing cell viability and proliferation, expression of neuronal markers, average neurite length, and direction of neuroblastoma N2A cells induced to differentiate on this novel support. In addition to the beneficial effect of the nanogrooved topography, a 30% increase was shown in the average length of neurites when differentiating cells were subjected to an electrical stimulation of a few microamperes for 6 h. The results reported here suggest a favorable effect on the neuronal development of the synergistic combination of nanotopography and electrical stimulation, supporting the use of NanoPEDOT in neural tissue engineering to promote physical and functional reconnection of impaired neural networks.

## Introduction

Axonal dysfunction and degeneration are associated with severe pathological conditions of central nervous system (CNS) diseases such as Parkinson’s disease, Alzheimer’s disease, amyotrophic lateral sclerosis, or can occur upon traumas, including brain injury, ischemic stroke, and spinal cord injury (SCI). For instance, upon SCI, the poor capability of endogenous physical and electrical reconnection of the CNS has been attributed to the progressive establishment of a nonpermissive trophic environment characterized by a paucity of neurotrophic growth factors and an abundance of axon growth inhibitory molecules.^1^ Unfortunately, the therapeutic options currently available for patients affected by SCI are limited to symptomatic treatments, encompassing the use of neuroprostheses and brain machine interfaces to stimulate supraspinal locomotor recovery, i.e., to support spare fibers to bypass the injury and reconnect the spinal cord.^2^ As axonal recruiting and growth is fundamental to restore connectivity and reestablish motor control after SCI, tissue engineering solutions such as 3D scaffolds and conduits have been extensively explored as physical bridges capable to support the adhesion, migration, and differentiation of neural cells (including glial cells, Schwann cells and neural stem cells), since they represent a topotactical guide for nerve regeneration.^3,4^ To further accelerate neurite outgrowth and axonal reconnection across the gap, scaffolds and conduits are commonly organized into hierarchical structures, including vessels, longitudinal or aligned fibers.^5^ In this regard, the role of biomaterial topography, and of anisotropic (grooves, fibers) and isotropic (pillars, nanowires, cones) structures capable of influencing neuritogenesis and outgrowth polarization, was extensively investigated, especially in vitro.^6−10^ Even though micro- or nanogrooves are among the simplest topographies conceivable, they result of particular interest as they resemble the extracellular matrix (ECM) architecture and in particular the organization of aligned radial glia and subventricular cells found in the developing brain.^11,12^ In this perspective, surface grooves can be envisioned as privileged tracks for the axon aiming to reconnect with other neurons on the other side of the lesion gap.^13,14^ In mammals, the typical size of a soma is on the order of 10 μm, the axon and dendrites diameter of about 1 μm and less in their distal part.^15^ Yet, it should not be surprising that, in general, platforms featuring groove dimensions in the 1–10 μm range provided a higher level of control on longitudinal axon growth compared to smaller or larger dimensions.^9,16^ Besides topographical cues, the possibility to enhance neuronal polarization and differentiation on electroactive biomaterials by the application of static or variable electric (magnetic) fields has been recently reported.^17,18^ Among electroactive biomaterials, conductive polymer-based composites and hydrogels are steadily gaining interest due to the possibility of combining in one single material high biocompatibility, ease of processability, softness and flexibility, and electrical properties.^19−21^ Remarkably, poly(3,4-ethylenedioxythiophene) poly(styrenesulfonate) (PEDOT/PSS) has been yet demonstrated to boost cell adhesion, proliferation and differentiation, finding application mainly in bone and neural tissue engineering.^17,22−25^ A particularly exciting aspect lies in the possibility to control neural stem cells differentiation on PEDOT/PSS by applying low-voltage stimulation trains of voltage pulses.^26−28^ Recently, some articles have demonstrated the possibility of fabricating by electrospinning polymeric blends based on conducting polymers to promote neural or myoblast cell differentiation under the application of electrical stimulation.^21,29−32^ However, randomly arranged mats of conducting nanofibers allowed only little or limited control over the axonal direction because of the stochastic distribution of the electrospun fibers. We instead believe that the possibility to handle multifunctional polymeric substrates, featuring regular topographical and electrical/magnetic guidance at once and easy integration into tissue engineering 3D constructs, would be highly desirable.

Here, we report for the first time a simple route for the fabrication of nanomodulated and conductive PEDOT/PSS substrates. We show that these substrates promote neuronal development in terms of neurite outgrowth, especially when electric stimuli are administered. These results hint at an innovative multimodal cue-supplying platform for neural tissue engineering aimed to actively accelerate neuronal development.

## Experimental Section

### Fabrication of the Nanomodulated PEDOT/PSS Substrate

Nanomodulated and flat PEDOT/PSS substrates were obtained by electrochemical deposition of PEDOT/PSS on the front and rear part, respectively, of a commercial polycarbonate (PC) compact disk (CD; pitch 1.5 μm, groove width ∼650 nm, ridge width ∼850 nm, ridge height ∼ 180 nm) upon metallization of the latter. Briefly, after peeling off the CD protective layer, the antistatic layer on the front part was removed by abundant rinsing in ethanol followed by 15 min of sonication in (1:1 v/v) ethanol/Milli-Q water solution. A thin gold film (∼30 nm) was then deposited on the surface of the by means plasma sputtering operating in argon using a 99.99% pure gold target (pressure ≈ 10^–1^ bar, time: 3 min, average current: 18 mA). For the electrochemical deposition of PEDOT/PSS, a solution of 0.01 M EDOT ethylenedioxythiophene (EDOT, Sigma-Aldrich, MO, USA) and 0.8% w/w poly(sodium 4-styrenesulfonate) (NaPSS, Sigma-Aldrich, MO, USA) was prepared. A large-area platinum mesh (30 × 15 mm^2^) and a standard Hg|Hg_2_Cl_2_ electrode were used as counter electrode and reference electrodes, respectively; the metalized PC substrates (flat or nanomodulated) were used as working electrode and contacted with silver conductive paste. The electrochemical polymerization was obtained by the chronocoulometry method, to allow a fine control of the amount of deposited charge (75 mC cm^–2^) and, therefore, on the final thickness of the PEDOT/PSS layer (∼50 nm).

### AFM Analysis

The surface topography of the gold-sputtered and electrodeposited PEDOT/PSS on either flat or nanomodulated PC substrates was investigated by AFM using a Park XE7 AFM system (Park System, Suwon, Republic of Korea) operated in tapping mode in air and at room temperature. Premounted silicon cantilever with Al backside reflective coating and typical tip curvature radius ca. 7 nm, elastic constant ca. 26 N m^–1^, and resonance frequency ca. 300 Hz (OMCL-AC160TS, Olympus Micro Cantilevers, Tokyo, Japan) were used. The line profile root-mean-square roughness (rms) of the different substrate and the thickness of the PEDOT/PSS coating were analyzed by both the Park System XEI software (Park System, Suwon, Korea) and Gwyddion freeware (version 2.61 http://gwyddion.net/).

### Electrochemical Characterization

Electrochemical properties of both flat and nanomodulated PEDOT/PSS substrates were evaluated by electrochemical impedance spectroscopy (EIS). EIS measurements in the range 1–10^5^ Hz were performed in saline solution (NaCl, 0.15 M) in a three electrode cell using standard Ag|AgCl (3 M KCl) as reference electrode, a large area platinum mesh (30 × 15 mm^2^) as counter electrode, and the substrate itself as working electrode.

### Electrical Characterization

Four-point probe measurements were used to extract sheet resistance values of both flat and nanomodulated PEDOT/PSS substrates.^33^ Briefly, four blunt stainless-steel probes were placed in contact with the sample surface at the vertices of a 1 × 1 cm^2^ delimited area. A constant value of current (0.1 mA) was forced for 10 s and then raised to the desired value of 0.5 mA using a two channel source-measure unit (Keysight, CA, United States). Measurements were performed in both normal and hydrated conditions which were obtained by soaking the sample in Milli-Q water for 1 h. Resistance (*R*) was extracted from Ohm’s law and used to calculate the sheet resistance (*R*_s_) as shown by the following equation^34^
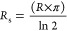


### Contact Angle Measurements

Water contact angle values were measured for all the substrates using a home-build contact angle measurement unit to collect images and ImageJ freeware (Rasband, W.S., ImageJ, U.S. National Institutes of Health, Bethesda, Maryland, USA, https://imagej.nih.gov/ij/, 1997–2018)^35^ to extract the angle value (averaged over different areas of at least three different samples for group).

### N2A Cell Culture

In order to evaluate the capability of nanomodulated PEDOT/PSS substrates to support neural cell growth and promote differentiation, we used a mouse Neuro2a (N2A) neuroblastoma cell line (Tema Ricerca, Bologna, Italy) as cell model, being a well-established line for studies of axon growth, neural differentiation, and future strategies for the treatments of nerve injuries. Before cellular studies, all substrates were sterilized in 70% v/v ethanol/water solution for 30 min, dried under laminar flow hood, and rinsed twice with sterile phosphate buffer saline (PBS pH 7.4). N2a cells were seeded at a density of 1 × 10^4^ cells/well on flat or nanomodulated PC and PEDOT/PSS-coated PC substrates put in 6-well culture plates (Corning Costar TC-Treated Multiple Well Plates, Merck Life Science S.r.l., Milan, Italy). Biphasic silicon rubber (Silicone RPRO 30) squared pools (1 cm × 1 cm) were fabricated on the surface to confine cells on the substrates. Cells were grown in 1 mL of Dulbecco’s modified Eagle’s medium (DMEM), containing 10% v/v fetal bovine serum (FBS), 200 μg/mL l-glutamine, 200 μg/mL penicillin/streptomycin. N2A culture wells were incubated at 37 °C with 5% CO_2_ and at approximately 90% relative humidity for 36 h.

### N2A Cell Viability

Through the generation of formazan as a result of a reduction oxidation process, the 3-(4,5-dimethyl-thiazol-2-yl)-2,5-diphenyltetrazolium bromide (MTT) assay (Thermo Fisher Scientific, Milan, Italy) was utilized to assess mitochondrial activity. In brief, cells were seeded onto each substrate condition as previously reported at a density of 1 × 10^4^ cells/well. At DIV1 and DIV6, 5 mg/mL of diluted MTT solution in PBS was added, then the mixture was incubated for 4 h at 37 °C with a CO_2_ (5%) atmosphere. The Formazan crystal produced during the experiment was dissolved by adding 100 μL of dimethyl sulfoxide (DMSO) to each well and the mixture was shaken for 45 min at 37 °C. Each sample was then transferred to a 96-well plate and a multimode plate reader spectrophotometer (VICTOR X4, PerkinElmer, Waltham, MA, USA) was used to measure the absorbance at 570 nm.

### N2A Cell Differentiation

After 36 h of incubation under the above conditions, each well was rinsed once with PBS and the medium was changed to a reduced amount of serum, combined with the addition of all-*trans* retinoic acid (atRA), (Thermo Fisher Scientific, Milano, Italy) in order to inhibit cell growth and promote neuronal differentiation. Specifically, the differentiation medium was composed of DMEM containing 1% FBS, 200 μg/mL l-glutamine, 200 μg/mL penicillin/streptomycin, and atRA 10 μM. Twenty-four hours of atRA stimulation was performed, followed by washing and fixing for DIV1. Up until the achievement of the DIV6, the complete stimulating medium was changed every 2 days.

### Analysis of Neurite Orientation and Average Neurite Length

In order to analyze neurite orientation, at DIV 1 and DIV 6 the cells were washed with PBS and fixed in 2.5% glutaraldehyde for 20 min, after which they were washed once more before proceeding with dehydration using ethanol at gradually increasing concentrations. Neurite outgrowth, in terms of neurite orientation and average length for cell, was quantified from optical micrographs at 20× magnification using the NeuriteTracer plugin of ImageJ,^36^ considering only well distinguishable and measurable neurites, aside from those at the edge. At neurite bifurcation points, a single neurite path was selected by following the longer branch of the extension. The number of neurite-bearing cells was calculated from at least three independent images obtained from nonoverlapping areas on each substrate.

### Electrical Stimulation Experiment

An ad-hoc measurement setup was ad hoc designed to allow DC stimulation of cells seeded on nanomodulated PEDOT/PSS substrates. Briefly, a six multiwell culture plate was modified so that each stimulation well was endowed with two electrodes (gold plate wires), being the terminals at which the stimulating bias was applied. Two of the six wells were used for the control samples (unstimulated group), thus they were not modified. Wires were connected to a multiplexer (outside the cell incubator) which was, in turn, connected to a two channel Keysight B2912A source-measure unit (Keysight, CA, United States) to apply a constant voltage to the samples of 1 V (generating an average current of 15 ± 4 μA during the stimulation experiment for all investigated substrates). Cells were electrically stimulated for 6 h, after having let them grow in undifferentiating medium for 36 h and then in differentiating medium for 24 h. At the end of the electrical stimulation, cells were further cultured for 18 h, then fixed and the neurite length measured. Stimulation time was defined based on the results of preliminary tests using smaller stimulation times (1–3 h) which, however, led to poorly appreciable differences in average neurite length compared to control samples.

### Confocal Immunofluorescence Analysis

Cells were fixed with 4% paraformaldehyde at 4 °C for 20 min and then permeabilized with 0.1% Triton X-100 for 5 min. After blocking with 2% bovine serum albumin (BSA) in pH 7.4 phosphate buffer saline (PBS) for 1 h, N2A cells were incubated at room temperature for 3 h with anti-β-III tubulin recombinant rabbit monoclonal antibody (Life Technologies, Monza, Italy), diluted 1:100 in 0.1% BSA in PBS. The cells were then incubated with Goat antirabbit IgG (H + L) Highly Cross-Adsorbed secondary Antibody, Alexa Fluor Plus 488 (Life Technologies, Monza, Italy) at 1:600 dilution in 0.1% BSA at room temperature for 45 min. Intracellular F-actin was detected with CellMask Deep red Actin Tracking stain (Life Technologies, Monza, Italy) incubated the cells at 1× concentration for 30 min at 37°. Nuclei were counterstained with 10 μg/mL Hoechst 33342 blue dye. Images were acquired with a Nikon A1 confocal laser scanning microscope.^37^

### SEM Analysis of Neural Cell Morphology

For SEM evaluations, cells cultured on substrate at different time points were fixed in 4% glutaraldehyde in phosphate buffer (pH 7.4), postfixed in 2% osmium tetroxide, dehydrated in an ascending series of alcohols, and dried with hexamethyldisilazane. Samples were then mounted on a metal stub and gold-sputter-coated (15 nm) with a Q150RS magnetron sputter (Quorum Tech, London, UK). ASEM ZEISS EVO40 XVP (Carl Zeiss NTS GmbH, Oberkochen, Germany) was used, operating at a 20 kV acceleration voltage.

### AFM Analysis of Differentiating Neural Cells

Differentiating N2A cells were investigated by AFM at different experimental times using the same microscope used for the characterization of film topography, operated in air and upon cell fixation (according to the same protocol used for the SEM analysis). Large area images (40 × 40 μm) were initially acquired to spot the whole cell; then decreasing scan size images were acquired to get additional insights about lateral dimension of neurite and axons and spot additional details such as morphology of the growth cones.

### Statistical Analysis Section

**Figure 1:**
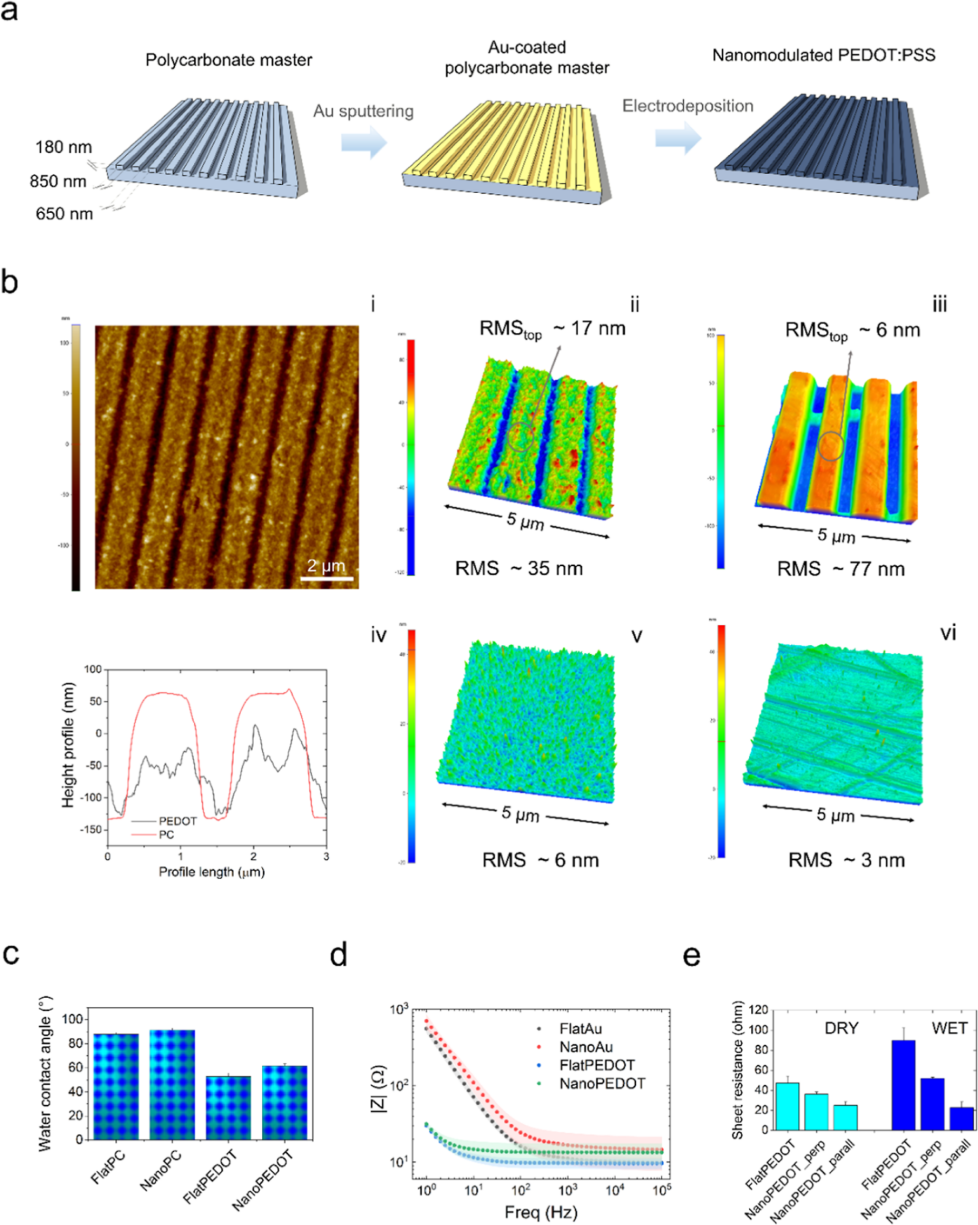
Fabrication and characterization of the NanoPEDOT. (a) Sketch of the fabrication process, encompassing metallization of a nanomodulated polycarbonate substrate, and subsequent PEDOT/PSS electrodeposition. (b) AFM analysis of the substrates investigated in this study: 2D topography image (i) and 3D rendering (ii) of NanoPEDOT; 3D topography of NanoPC (iii), height profiles NanoPEDOT and NanoPC (iv); 3D rendering of FlatPEDOT (v) and FlatPC (vi). (c) Water contact angle and (d) EIS spectra of the investigated samples (*n* = 3). (e) Sheet resistance under dry and wet conditions of FlatPEDOT and NanoPEDOT (*n* = 3). In (c) significant difference (*p* < 0.05) exists among all the investigated samples; however, for clarity no marks have been reported in both the plots.

**Figure 2:**
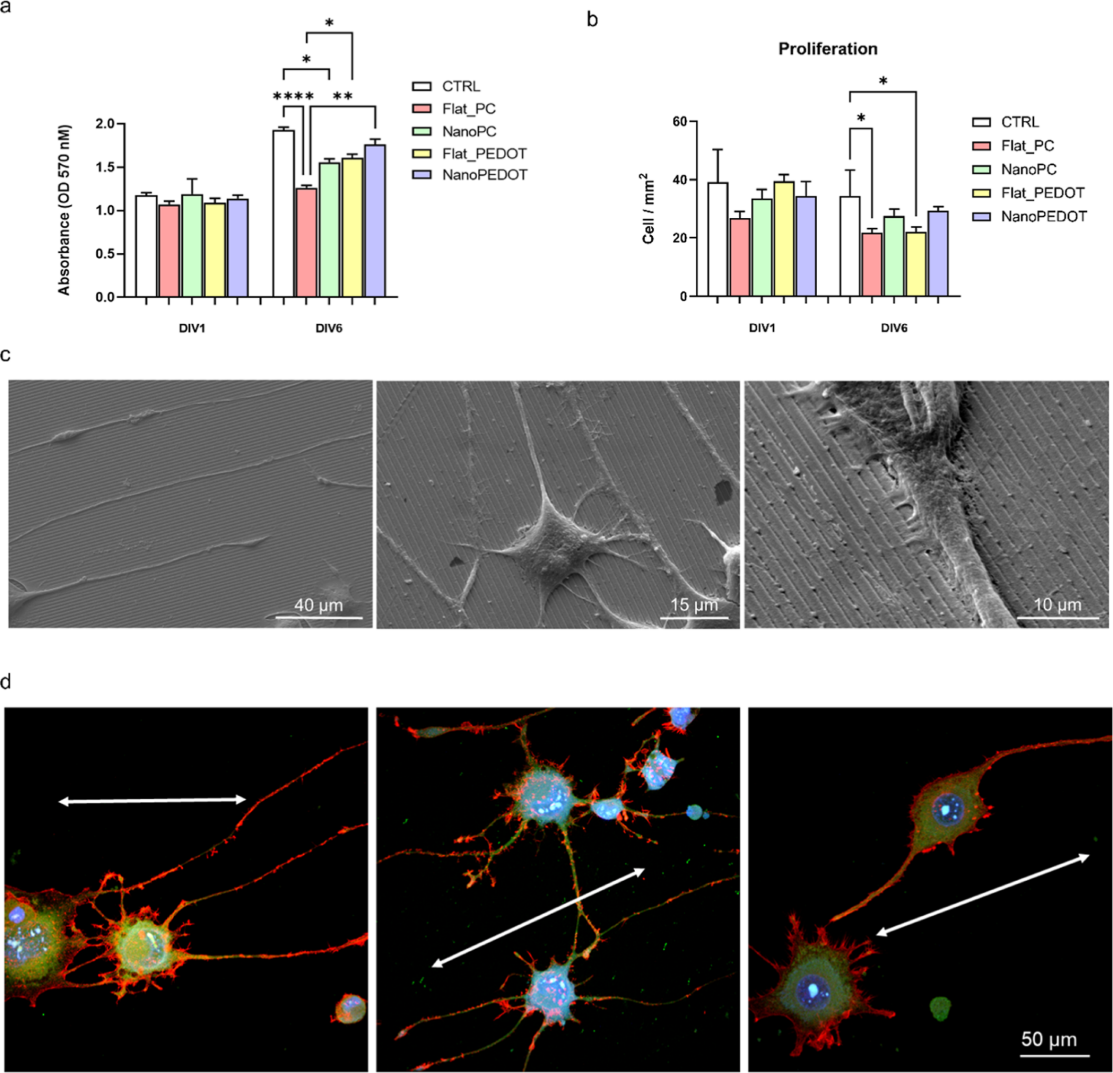
Evaluation of N2A cell differentiation on NanoPEDOT. Results from (a) MTT and (b) proliferation tests. *, **, **** refer to *p* < 0.05, *p* < 0.01, and *p* < 0.001, respectively (*n* = 3). (c) Scanning electron microscopy (c) and immunofluorescence (d) images of N2A cells at DIV 6 on NanoPEDOT. In (d), the following neuronal markers are shown: β-III tubulin (green), intracellular F-actin (red), and Hoechst 33342 (blue); the double-ended arrows are a guide-for-the-eye to show the main direction of the pattern compared to the direction of the axons.

**Figure 3:**
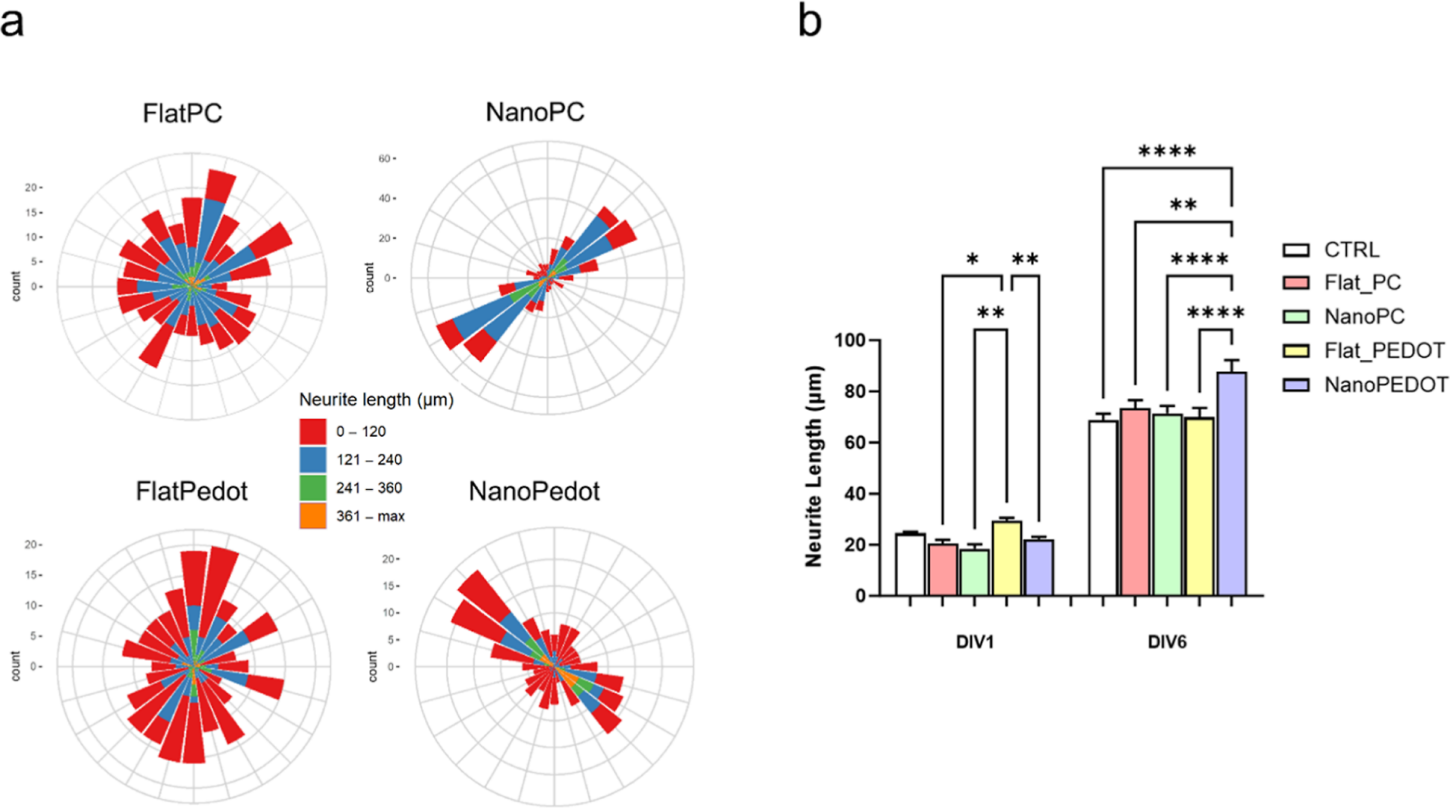
Analysis of cell neurite polarization. (a) Polar plots showing the angular distribution of neurites with respect to the main pattern direction (indicated by the dotted line) at DIV 6 (*n* = 3). Each sector corresponds to a range of 15°. (b) Average neurite length per cell at DIV1 and DIV6 (*n* = 3).

**Figure 4:**
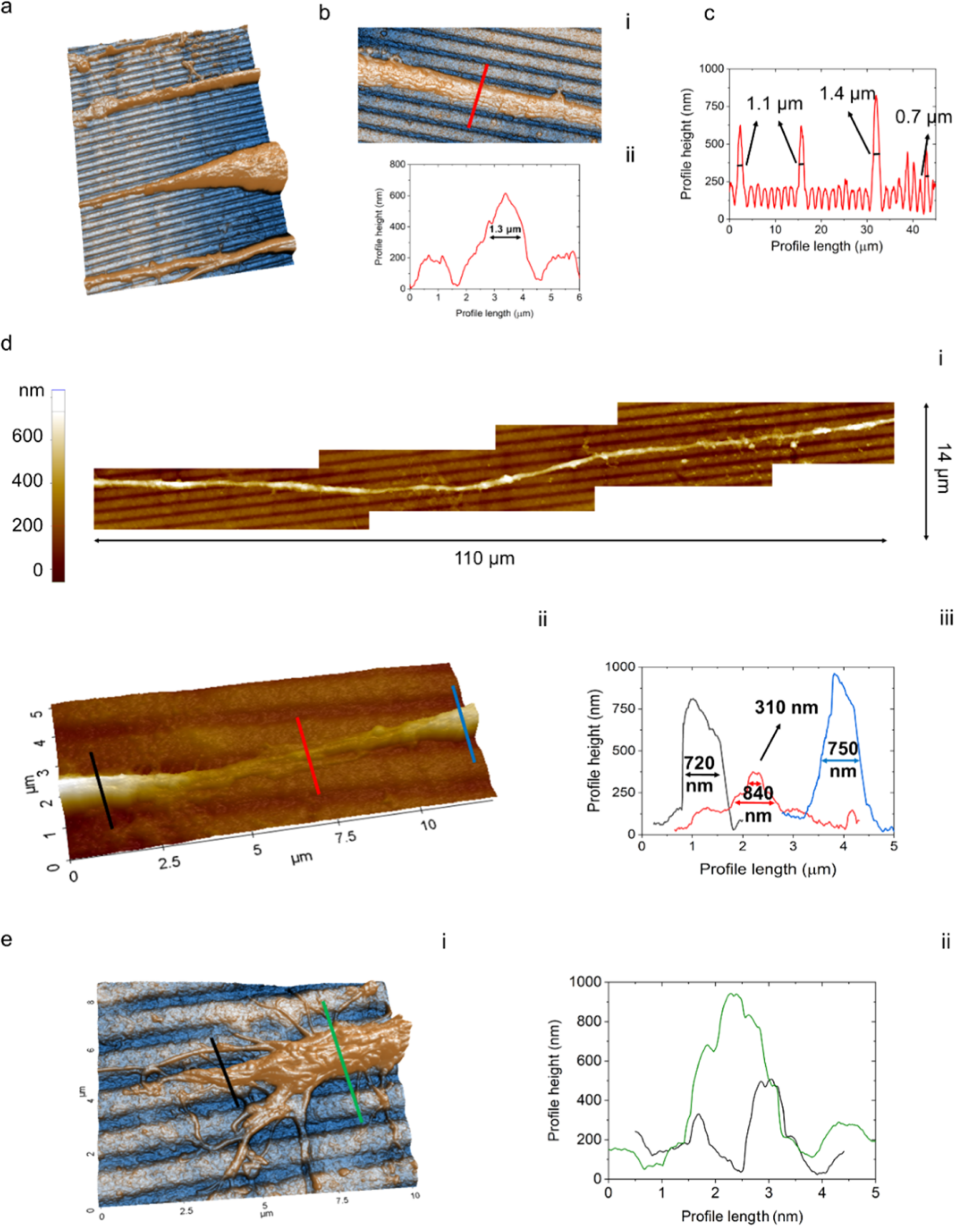
AFM analysis of neurite morphology on NanoPEDOT (DIV6, *n* = 3). (a) (45 × 30 μm) 3D topography image of parallel neurites from different cells. (b) (20 × 10 μm) topography image (i) and height profile (ii) of a portion of axon. (c) Line profile from (a), showing the presence of both axonal and smaller neurite processes. (d) Combination of four different AFM scans (i) enabling one single neurite to follow up to more than 100 mm of length; (ii) detail from (i) showing the squeezing of the neurite inside the nanogroove, and (iii) relative height profiles. (e) 3D rendering (i) and relative height profiles (ii) of a growth cone.

**Figure 5:**
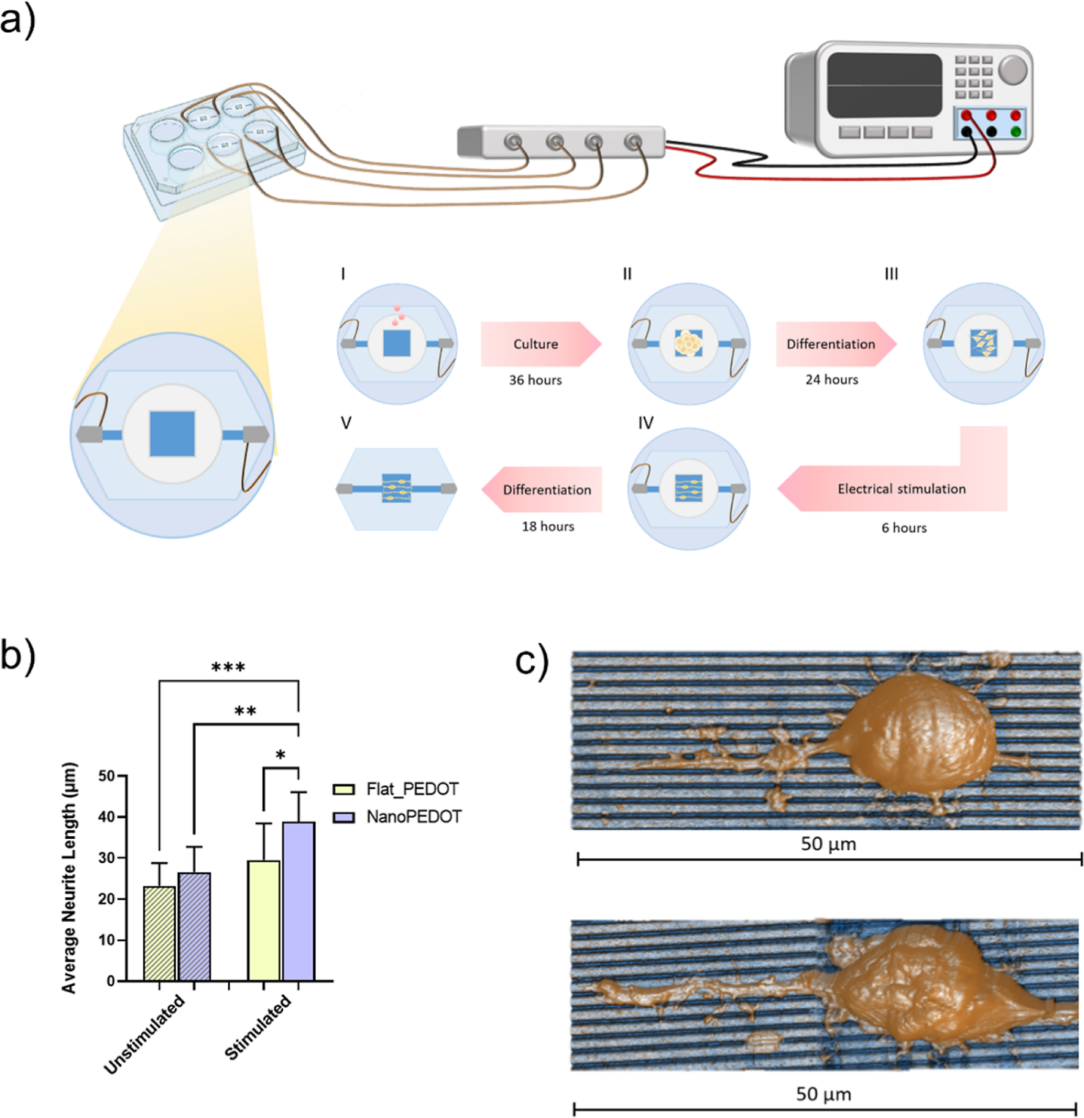
Electrical stimulation of neural cells on nanoPEDOT. (a) Sketch of the setup used for the electrical stimulation experiment. (b) Average neurite length of N2A differentiating on NanoPEDOT and FlatPEDOT with and without electrical stimulation (*n* = 3). (c) Representative AFM images of neurites sprouted from stimulated (top) and nonstimulated (bottom) N2A cells on NanoPEDOT.

All data from biological experiments (shown in Figures 2–5) were analyzed by Graphpad software (GraphPad, version Prism 9.5.1, GRAPHPAD 2365 Northside Dr., San Diego, CA, USA, 2102) applying one-way analysis of variance (ANOVA) followed by Tukey’s post-test. Level of significance was set at *p < 0.05. Data about the wettability and sheet resistance shown in Figure 1 were analyzed with Origin 2016 Software (OriginLab Corporation, Northampton, MA, USA). Level of significance was set at *p < 0.05. All data were expressed as means ± standard deviations of experiments carried out in triplicate (*n* = 3).

## Results and Discussion

### NanoPEDOT Substrates

Highly homogeneous nanomodulated PEDOT/PSS (NanoPEDOT) substrates were obtained via the simple fabrication process sketched in Figure 1a. Nanomodulated PC (NanoPC) substrates featuring submicron nanogrooves or unmodulated PC (FlatPC) substrates were used as templates for the electrodeposition of the PEDOT/PSS layer, after the PC substrate was sputtered with a gold thin film.

We opted for the deposition of a very thin and homogeneous layer of PEDOT/PSS (∼50 nm), in order to limit the concealing of the underlying substrate topography, which is the effector instrumental for guiding cell differentiation. As local topography at micro- and nanoscale is a well-established key parameter affecting reversible and unreversible protein adhesion before and after cell attachment,^38−40^ surface rms was preliminarily assessed by AFM analysis (Figure 1b). The surface of ridges in the NanoPEDOT [Figure 1b(i)] appeared rougher compared to that of NanoPC, being characterized by the presence of nanosized grains (lateral size 20 ÷ 50 nm), typical of electrodeposited PEDOT/PSS thin films.^41,42^ Such granular morphology led to a considerable rms increase on the top of the ridges for NanoPEDOT [rms ∼ 17 nm, Figure 1b(ii)] compared to NanoPC [rms ∼ 6 nm, Figure 1b(iii)]. However, as the conductive polymer was deposited not only on the top of the ridges but even in the patterned grooves, both an average decrease of the groove depth (from 180 nm of NanoPC to ∼75–120 nm for NanoPEDOT) and an average increase of the ridge size (from 850 nm of NanoPC to ∼950–1000 nm for NanoPEDOT) were observed. Overall, the deposition of the PEDOT/PSS thin layer reduced the roughness of the samples compared to that of bare nanomodulated polycarbonate, despite the former being endowed with a locally larger (nano)roughness. As expected, the unmodulated samples (FlatPEDOT and FlatPC) showed much lower rms values, being in the order of a few nanometers [Figure 1b(v,vi)]. Surface wettability is another surface property capable to promote or not cell adhesion on a given material.^43−45^ In this study, PEDOT/PSS-coated samples (being nanomodulated or not) resulted moderately more hydrophilic than the corresponding uncoated substrates (Figure 1c), likely due to the electrostatic nature of the conducting polymer which favors the adhesion and spreading of water molecule on its surface compared to the mildly polar polycarbonate surface.^46^ As expected by the higher rms values, nanomodulated surfaces (PEDOT or PC) were found to be slightly more hydrophobic than the corresponding flat samples.

### Electrochemical and Electrical Characterization

The impedance spectra of the gold-coated and PEDOT/PSS-coated polycarbonate substrates are shown in Figure 1d. The deposition of the thin PEDOT/PSS layer enabled a two-orders-of-magnitude impedance drop compared to the respective gold-coated PC substrates in the low-frequency region, with |*Z*| values dropping from ∼700 to ∼30 Ω at 1 Hz. Being typical of PEDOT coatings, such a notable reduction was accompanied by a large broadening of the frequency independent region, with the cutoff frequency shifting from 10^2^ ÷ 10^3^ Ω to below 10 Ω, which suggests optimal charge transport between the underlying gold layer and the upper PEDOT film.^47,48^ However, the similar impedance behavior for PEDOT-coated and uncoated gold substrate in the high frequency region indicated that in this domain the resistive behavior was overshadowed by the resistance of the gold film, likely due to the very low thickness of the conductive polymer layer. Finally, no statistical differences were detected between the impedance of the nanomodulated and flat substrates. Results of in-plane sheet resistance analysis obtained from four-point probe electrical measurements for NanoPEDOT and FlatPEDOT are reported in Figure 1e. Sheet resistance values, in line with the literature ones,^49,50^ were higher for FlatPEDOT than those of NanoPEDOT, both in air or under wet conditions. In particular, sheet resistance measured along the main pattern direction was slightly lower than the one measured perpendicular to the grooves. We speculate that the reason for these results could lie in a preferential charge transport pathway for charge carriers on the nanomodulated film (especially when current is measured along the same direction of the grooves) compared to the isotropic one. Finally, measurements carried out on samples soaked in water highlighted higher sheet resistance values for FlatPEDOT and NanoPEDOT_perp, whereas the conductivity of NanoPEDOT_paral seemed to be not affected by the presence of the liquid medium.

### Effect of Nanomodulated PEDOT/PSS on Neural Cell Viability and Differentiation

N2A cells were induced to differentiate on NanoPEDOT and on the other investigated groups (FlatPEDOT, NanoPC and FlatPC) including a control cell (Petri dish). Cell viability, proliferation, and neurite outgrowth upon the presence of the PEDOT layer and/or the nanotopography were addressed (Figures 2 and 3). Viability of N2A cells was generally increased from DIV1 to DIV6 and was maximum for cells differentiating on NanoPEDOT compared to the other groups, similar to what was observed on the control group (Figure 2a).

Slightly higher viability was steadily observed for cells differentiating on nanomodulated samples compared to flat ones, to be ascribed to the positive effect of the higher local surface roughness and area on cell behavior. Due to the intrinsic low biocompatibility of polycarbonate compared to the other investigated groups, those based on PC markedly showed lower viability. As expected, the proliferation-differentiation switchover medium causes a reduction in the trend of cell proliferation over time (Figure 2b). Indeed, the proliferation-differentiation switches are caused by serum depletion combined with atRA addiction. As is common knowledge, serum contains lipoprotein and peptide growth factors that encourage cell proliferation; however, serum withdrawal results in a temporary restriction in the availability of cells. Furthermore, the typical role of atRA is to stop proliferation in order to act as a differentiation-inducing agent.^51^

Representative SEM images of N2A cells differentiating on NanoPEDOT are shown in Figure 2c. As can be observed, the cells’ axons and longest neurites grow following the main direction of the pattern, while shorter neurites and minor filopodia sprout from the neurons’ soma and grow along all directions, ensuring a tight anchoring to the substrate. In general, cell axons and longest neurites were found to accurately follow the longitudinal pattern, whereas shorter or minor filopodia sprouted from a neuron-like star-shaped soma to provide better cell anchoring to the substate. On the contrary, a randomly distributed outgrowth of neurites and axons was found, as expected, on unmodulated PEDOT/PSS substrate (Figure S1). Immunofluorescence analysis confirmed the advanced degree of differentiation of N2A cells cultured on NanoPEDOT into neurons by spotting the high expression of typical neuronal markers such as β-III tubulin, clearly appreciable in both minor (small neurites and filopodia) and longer (axons) cell protrusions (Figure 2d). More immature expression of neuronal markers was instead found at the same experimental time on cells differentiating from the other investigated samples (Figure S2). Average direction of the cell neurites is reported in Figure 3a. Independently from the substrate surface chemistry, the presence of the pattern establishes a preferred growth direction (polarization) for neurites unlike the unmodulated surfaces or control groups (Figure S3). These data confirm what previously reported about the effect of nanosized grooved on neurite outgrowth direction.^9,16^ However, for NanoPEDOT, it can be noticed that a not negligible population of relatively short neurite is oriented almost perpendicular to the longitudinal pattern direction. Such phenomenon, termed as “neural bridge”, has been already reported in literature for similar patterns^16,52^ and, in this work, can be ascribed to the extensive presence of sprouting processes firmly attaching the cell to the PEDOT/PSS layer. This finding, together with the observation of a significantly larger neurite length developed by cells on NanoPEDOT compared to the other substrates and FlatPEDOT in particular (+25% at DIV6, Figure 3b), only confirms how much the growth and differentiation of cells are promoted on this nanostructured conductive polymeric layer. At DIV6, neurites of cells grown on NanoPEDOT were found to be ≈25% longer than those of cells cultured on FlatPEDOT (Figure 3b). In contrast, no significant effect on neurite elongation was observed in cells cultured on NanoPC compared to FlatPC. Hence, it is possible to state that the nanostructured PEDOT/PSS substrate strongly promotes both the growth and the differentiation of N2A cells.

The optimal affinity of cells for PEDOT/PSS has been reported in a number of papers and is commonly attributed to its surface charge, rather than to its relatively rough and soft surface.^26,53^ In particular, proposed models for PEDOT/PSS microstructure, encompassing the presence of larger, negatively charged, PSS shells wrapping around smaller, positively charged, PEDOT domains,^54,55^ well account for an overall negative surface charge of the polymer which has been postulated as fostering fast and stable accommodation of adhesion proteins and, in turn, cell spreading and colonization of the substrate.^8,24,26,56^ Overall, our data support the hypothesis that combination of nanotopography and local nanoroughness with the particularly well suited surface chemistry of PEDOT/PSS, synergistically cooperate into promoting neuronal polarization and accelerating neurite development, especially if compared to unmodulated PEDOT/PSS (FlatPEDOT) or uncharged nanomodulated (NanoPC) substrates.

### AFM Analysis of Neurites

AFM is an invaluable tool in biophysics to simultaneously get qualitative and quantitative topographical information compared to optical or electronic microscopy at the micro- and nanoscale.^57,58^ Here, we investigated by AFM the characteristic dimensions of sprouted neurites and their intimate interaction with the nanotopography of the conductive substrate (Figure 4). We investigated cells at an intermediate differentiation stage (DIV 6) in order to be sure to identify developed axons (as observed from optical and scanning electron microscopy), other than minor neurites (i.e., dendrites). We excluded from this analysis the extremely short processes close to the cell soma. By focusing on sample areas showing several protrusions from different cells such as the one reported in Figure 4a, we were able to spot the presence of a distinct population of relatively large processes with later size of (1.3 ± 1) μm [Figure 4b(i,ii)]. Being the latter the larger size found in the sample, in good agreement with previous optical and AFM investigations,^59−61^ it was quite easy to assign this population to that of mature axons. This fact hints that geometrical matching between axonal diameter and pitch dimension (≈1.5 μm) played a pivotal role in directing neurite growth on this kind of pattern. Average size of smaller neurites was instead found to fall under a broader distribution curve ranging approximately from 0.7 to 1.1 μm (Figure 4c). We assigned this more heterogeneous population to both dendrites and immature axons, making it at this stage impossible to distinguish between the two.

Interestingly, AFM analysis allowed us also to spot quite uncommon cell protrusions such as the one shown in Figure 4d. Here, the topography of an average size neurite, acquired by moving the scan area along the direction of the neurite for a total length of 110 μm and stitching the AFM images together, is shown [Figure 4d(i)]. As can be appreciated, the neurite follows the direction of the grooves quite conformally even though its diameter (≈0.75 μm) does not match the size of the ridge (1.0 ÷ 1.1 μm) or the groove (0.4 ÷ 0.5 μm). Even more interestingly, the neurite was found to squeeze(deform) in several places several times in the scanned area to be able to allocate itself within the depth of the groove [Figure 4d(ii,iii)]. Taken together, the AFM data corroborate the high suitability of the selected pattern dimensions for the alignment and direction of both axonal and smaller neurites.

### Analysis of Growth Cones on NanoPEDOT

Growth cones can be found on the distal part of a growing neurite; they are structures that continuously change their morphology for the purpose of probing the surroundings and determining the direction of growth of the neurite which, in the absence of instructional cues, proceed along a relatively straight path.^62^ Growth cone direction hence relies on tireless synthesis and disruption of actin filaments that constitute the major portion of the filopodia, with the latter being continuously expanded and retracted. In SI_Movie_GrowthCone1 and SI_Movie_GrowthCone2, the highly dynamic process of growth cone advancement on NanoPEDOT on a time scale of 4 h can be appreciated. Remarkably, the growth of the cone does not simply proceed along the main direction guided by the nanotopography as one may expect. It is instead the result of a sort of “trial and error” process, aimed to span around the surrounding environment on an ≥180° spectrum. The fact that the axon keeps on growing on the main pattern direction rather than jumping from one lane to another or even changing direction can be ascribed to the establishment of privileged tensional forces between the cell and the nanogrooved substrate along the main pattern direction, as previously reported for other nanomodulated/nanopatterned systems.^63−65^ In SI_Movie_Connection, one neurite can be observed deviating from the main direction of the pattern to establish connections with another nearby neurite belonging to a second cell. In this case, the presence of soluble chemotactic cues expelled by cells in their proximity, can be invoked to explain this behavior. The presence of chemical gradients can be speculated to lead to the decision to make a neurite abandon the main direction pattern, cross the nanogrooves, and create a synaptic connection with the nearby neurite form another cell.

Along with axons, dendrites, soma, and synapses, growth cones also have been the subject of investigation with AFM, even if the literature in this field is still at its infancy.^61,66−68^ A 3D AFM image of a growth cone of an axon developing along the NanoPEDOT is shown in Figure 4e(i) together with some profiles tracked on different areas of the cone [Figure 4e(ii)]. AFM analysis allowed us to spot several small diameter (200 ÷ 300 nm) filopodia appointed to span the surroundings and search for the preferred way to further trigger the axonal growth. The fact that the larger cone terminal bundle (∼600 nm of diameter) is on the top of the ridge and in the frontal part of the cone, together with the presence of bundles of microtubules infiltrating the growth cone (typical of an active cone^68^) leads us to postulate that the axon investigate in this image will likely keep on growing along that direction during the subsequent growth phase.

### Evaluation of Neurite Length upon Electrical Stimulation

To evaluate the advantage of handling a tailored nanomodulated surface and the possibility to electrically stimulate neural cells at the same time, we applied the stimulation protocol shown in Figure 5a to N2A cells cultured on both NanoPEDOT and FlatPEDOT. Cells underwent static mild electrical stimulation (≈15 μA) for a short period of time (6 h) during their differentiation process. At the end of the stimulation period, cells were let to further differentiate for additional 18 h. Notably, we found a remarkable +33% increase of the average neurite length for cell differentiating on NanoPEDOT when cells were electrically stimulated compared to nonstimulated ones, supporting the hypothesis of beneficial effects of stimulation on neuronal polarization, as previously demonstrated by others using different substrate materials or cell types.^27,69^ Even more interestingly, under stimulation, cells were found to sprout significantly longer (+24%) neurites when cultured on NanoPEDOT than on FlatPEDOT, clearly underlying the beneficial effect of the coupled approach (i.e., nanotopography and electrical stimulation).

Finally, it should be noted that data concerning neurite length obtained from the stimulation experiments cannot be directly overlapped with those shown in Figure 3, mainly due to the different protocols used (especially regarding the different experimental time at which neurite length was evaluated). In particular, we chose to electrically stimulate N2A cells during their early differentiating stage in the attempt to maximize the possible gain in average neurite length. However, it can be inferred from Figure 5b, without fear of falling into overly speculative deductions, that coupling the effect of nanomodulation with that of electrical stimulation leads to a net increase in average neurite length compared with the two decoupled single approaches. Thus, we conclude that the effects, viz. nanopatterned grooves and electrical stimulation act synergically to produce a larger elongation of aligned neurites on the surface, with formation of synaptic contacts and enhanced spreading of the soma of the neuronal cells.

## Conclusions

In this study, we successfully fabricated the PEDOT/PSS substrate showing a nanomodulated and nanostructured topography, able to accelerate and direct neuronal polarization. Neurite length could be enhanced further by the electric stimulation of neuronal cells during differentiation. This study emphasizes the significance of synergically integrating both topographical and electrical cues in the functional active material, here being a polymer, and thus easily integrated into advanced 3D tissue engineering solutions. It should be noted that, in this work, a nonbiodegradable substrate such as polycarbonate was used to demonstrate the proof-of-concept of our idea. However, for real transposition to the in vivo scenario, it will be necessary to use and integrate PEDOT/PSS into the 3D constructs either alone (e.g., as a 3D printed hydrogel) or grown on a biodegradable and biocompatible substrate.

## Supporting Information Available

SEM images of N2A cells on FlatPEDOT at DIV 6. Confocal immunofluorescence images of N2A cells on FlatPC at DIV1, NanoPC at DIV1, and NanoPC at DIV3 (n=3). Analysis of cell neurite polarization on control groups (petri dish) at DIV 1 and DIV 6 (n=3) (PDF)
This movie shows a growth cone exploring the surroundings on NanoPEDOT (MP4)
This movie shows some processes of growth cone advancement on NanoPEDOT (MP4)
This movies shows two neurites establishing connections on NanoPEDOT (MP4)
