# Supplementary material for: Synergy of Nanotopography and Electrical Conductivity of PEDOT/PSS for Enhanced Neuronal Development
Source: ACS Appl Mater Interfaces. 2023 Dec 13;15(51):59224–35. doi: 10.1021/acsami.3c15278 (PMC10755694; doi:10.1021/acsami.3c15278)
Supplement: Supplementary file 1 — am3c15278_si_001.pdf [file am3c15278_si_001.pdf]

Supporting information file

Synergy of nanotopography and electrical conductivity of PEDOT: PSS for enhanced neuronal development

Michele Bianchi[†, §, *], *Sonia Guzzo*[¥,§], *Alice Lunghi*[¥,§], *Pierpaolo Greco*[¥], *Alessandra Pisciotta*[°], *Mauro Murgia*[§, ǁ], *Gianluca Carnevale*[°], *Luciano Fadiga*[¥,§,#], *Fabio Biscarini*[†,§,#].

[†] *Department of Life Sciences, Università degli Studi di Modena e Reggio Emilia 44125 Modena, Italy.*

[§] *Center for Translational Neurophysiology of Speech and Communication, Istituto Italiano di Tecnologia 44121 Ferrara, Italy.*

[¥] *Section of Physiology, Università di Ferrara 44121 Ferrara, Italy.*

[°] *Department of Surgery, Medicine, Dentistry and Morphological Sciences with Interest in Transplant, Oncology and Regenerative Medicine, Università di Modena e Reggio Emilia 44125 Modena, Italy,*

// Istituto per lo Studio dei Materiali Nanostrutturati (ISMN-CNR) 40129 Bologna, Italy

# The authors equally contributed.

* correspondence to: michele.bianchi@unimore.it

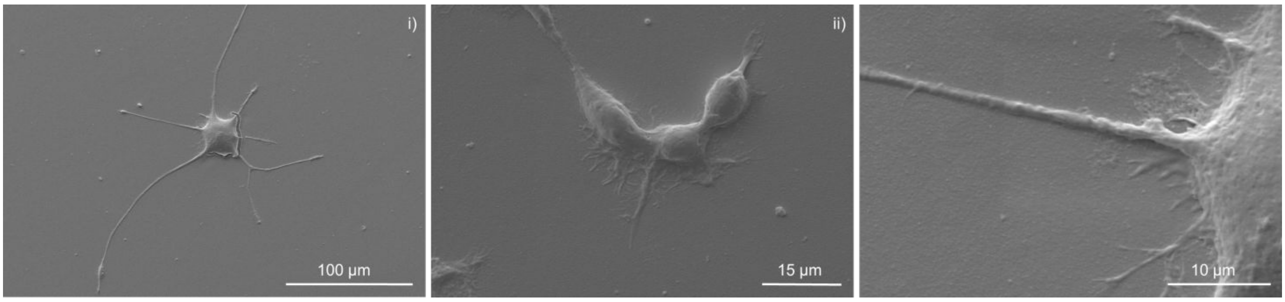

Figure S1. SEM images of N2A cells on FlatPEDOT at DIV 6.

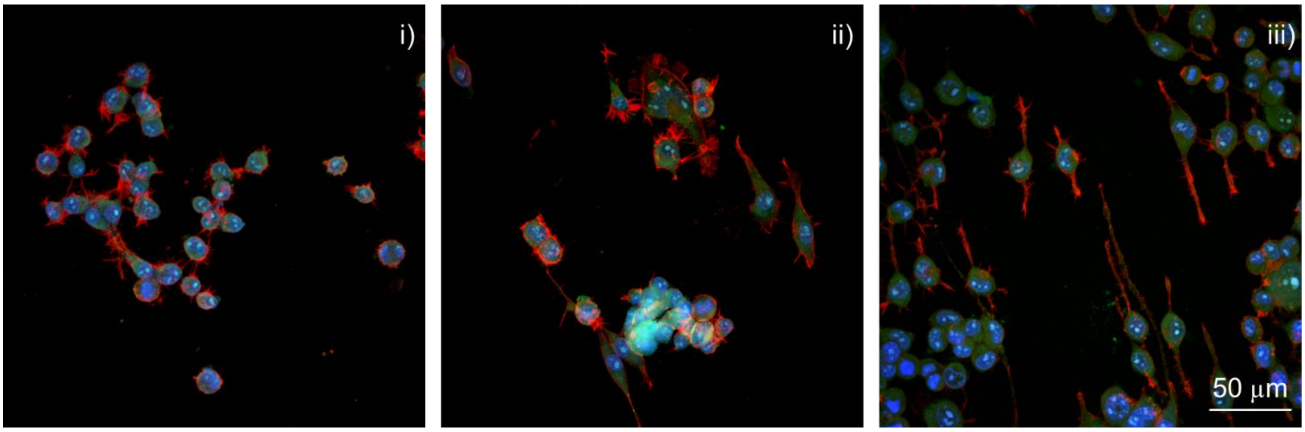

Figure S2. Confocal immunofluorescence images of N2A cells on (i) FlatPC at DIV1, (ii) NanoPC at DIV1 and iii) NanoPC at DIV3 (n=3). Cells were stained against β-III tubulin (green) and Intracellular F-Actin (red). Nuclei were counterstained with DAPI (blue).

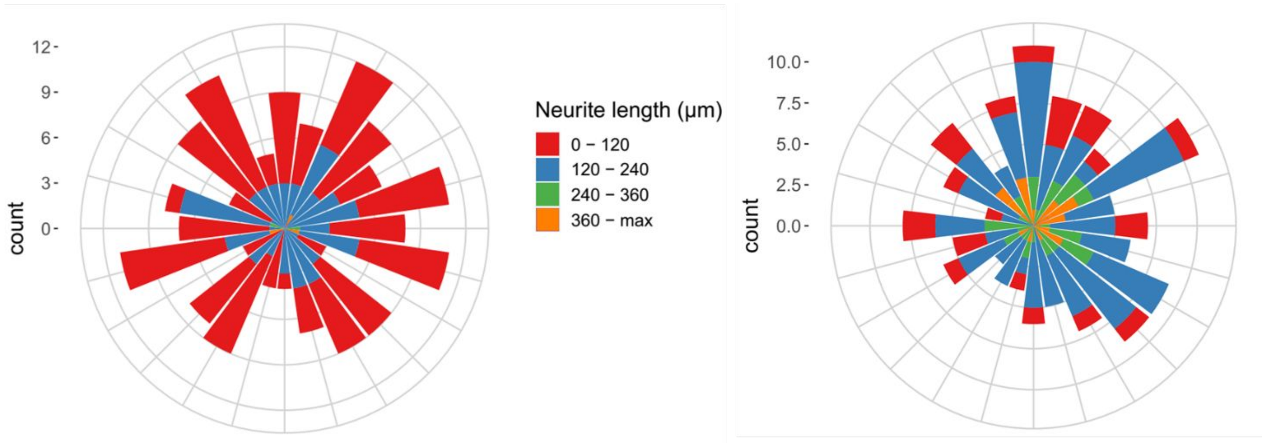

Figure S3. Analysis of cell neurite polarization on control groups (petri dish) at DIV 1 a) and DIV 6 (n=3).
